# Mechanosensitive ion channel Piezo1 modulates the response of rat hippocampus neural stem cells to rapid stretch injury

**DOI:** 10.1371/journal.pone.0323191

**Published:** 2025-05-13

**Authors:** Emanuele Mocciaro, Madison Kidd, Kevin Johnson, Elizabeth Bishop, Kathia Johnson, Ya Ping Zeng, Cristiana Perrotta, Maria-Adelaide Micci

**Affiliations:** 1 Gene Expression Regulation Unit, San Raffaele Scientific Institute, Milan, Italy; 2 Department of Biomedical and Clinical Sciences, Università degli Studi di Milano, Milan, Italy; 3 Department of Anesthesiology, University of Texas Medical Branch, Galveston, Texas, United States of America; Indiana University School of Medicine, UNITED STATES OF AMERICA

## Abstract

Traumatic brain injury (TBI) is one of the primary causes of long-term brain disabilities among military personnel and civilians, regardless of gender. A plethora of secondary events are triggered by a primary brain insult, increasing the complexity of TBI. One of the most affected brain regions is the hippocampus, where neurogenesis occurs throughout life due to the presence of neural stem cells (NSC). Preclinical models have been extensively used to better understand TBI and develop effective treatments. Among these, rapid stretch injury has been used to mimic the effect of mechanical stress produced by a TBI on neurons and glia *in vitro*. In this study, we aimed to determine the impact of rapid stretch on the viability, proliferation, and differentiation of NSC isolated from rat hippocampus (Hipp-NSC) and to determine the role of the stretch-activated ion channel Piezo-1 in modulating their response to mechanical stress. We found that while rapid stretch (30 and 50 PSI) reduced Hipp-NSC viability (measured as a function of LDH release), it did not change their proliferation and differentiation potentials. Interestingly, rapid stretch in the presence of a selective Piezo-1 inhibitor, GsMTx4, or Piezo1 targeting siRNA, directed Hipp-NSC differentiation toward a neurogenic lineage. Additionally, we found that inhibiting Piezo1 with the addition of a rapid stretch injury increased the expression of miRNAs known to regulate neurogenesis. This work uses a novel approach for studying the effect of mechanical stress on NSC *in vitro* and points to the critical role the stretch-activated ion channel Piezo-1 has in modulating the impact of TBI on hippocampal neurogenesis.

## Introduction

Traumatic Brain Injury (TBI) is a forceful episode that causes devastating brain damage, leading to morbidity and high mortality worldwide [1]. In addition to the primary insult, TBI triggers many secondary events such as inflammation, increased blood pressure, neuronal loss, and necrosis that collectively contribute to the complexity of this brain illness [2]. The primary cause of TBI is the mechanical force that leads to brain damage and neuronal death and, in the most severe cases, microvasculature disruption and blood-brain barrier failure [3].

The hippocampus is one of the most vascularized areas of the brain and one of the brain regions most susceptible to TBI-induced impairments in cognitive function and memory [4]. Moreover, the hippocampus is one of the two brain regions where neurogenesis occurs throughout life due to the presence of neural stem cells [5]. Neurogenesis is a tightly regulated multistep process that is affected by TBI [6–9]. Although increased proliferation of neural stem cells in the hippocampus has been reported to occur after a trauma to the brain, few progenitor cells reach the final stage of differentiation and become mature neurons able to integrate into the hippocampal circuitry [10]. In addition, we and others have reported that, following a TBI, newly generated neurons ectopically migrate in the hilus [11]. This not only contributes to the post-TBI onset of seizures and epilepsy but also the depletion of the pool of progenitor cells, resulting in the inability of the brain to recover from TBI [12,13]

Evidence in the literature shows how different experimental TBI models recapitulate some of the characteristics of human TBI with diverse consequences on neurogenesis [14]. For example, moderate to severe TBIs impair neuronal activity and metabolism, reducing hippocampal neurogenesis, while mild TBIs increase neurogenesis or only moderately perturb it [10,15]. It follows that after brain trauma, the brain’s ability to recover and replace damaged neurons is highly variable.

Most TBI research has focused on curing or preventing the secondary effects to ameliorate the patient’s prognosis [16]. However, as of today, there is no cure or effective therapeutic options available to TBI patients [17]. While several animal models of TBI are available to characterize the complexity of the secondary injury, the effects of the initial mechanical force at the cellular level are difficult to study [18].

Stretch-activated ionic channels are responsible for transducing mechanical stimuli from the environment [19]. Piezo1 is one of the most abundant stretch-activated ion channels expressed in neural stem cells and has been shown to play a pivotal role in their lineage determination [20]. Indeed, pharmacological inhibition of Piezo1 suppresses neurogenesis and induces astrogenesis [21]. While stretch injury models have been used to study the effect of axonal injury and mechanical force on mature neurons and glia *in vitro*, no information is available on the impact of mechanical stress on hippocampal neural stem cells [22].

In this work, we tested the effect of rapid (50 ms) stretch injury (30 PSI and 50 PSI) on the viability, proliferation, and differentiation of neural stem cells isolated from the hippocampus of adult rats (Hipp-NSC). Moreover, we tested the effect of inhibiting the stretch-activated ion channel Piezo1 using GsMTx4, a peptide isolated from the tarantula venom, or Piezo1 targeting siRNA on neurogenesis in the presence or absence of mechanical stress.

## Results

### Hipp-NSC grown on BioFlex silicone plates maintain their stem cell phenotype

**Fig 1:**
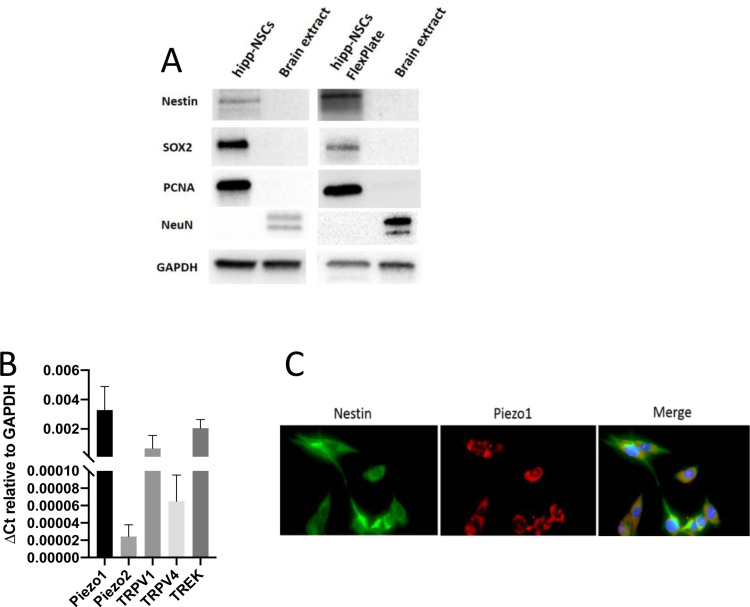
Hipp-NSC cultured on BioFlex plates maintain their stem cell phenotype and express mechanoreceptors. (A) Western blotting showing the expression of stemness markers (Nestin and Sox2), proliferation markers (PCNA), and the absence of the neuronal marker NeuN in both Hipp-NSC in suspension and Hipp-NSC cultured on BioFlex plate. GAPDH: loading control. (B) qRT-PCR showing the relative mRNA expression of the mechanoreceptors Piezo1, Piezo2, TRPV1, TRPV4 and TREK in Hipp-NSC (N = 8). (B) Immunofluorescence showing the expression of Piezo1 protein in Hipp-NSC. Nestin: marker of stemness. DAPI: nuclei.

Extracellular substrates are known to affect stem cells’ ability to proliferate and differentiate [23]. We first tested whether Hipp-NSC cultured on the silicon surface of the BioFlex plates maintained their stem cell phenotype. Western blot analysis revealed that Hipp-NSC grown on BioFlex plates in proliferation media for four days express the stemness markers nestin and Sox2 but don’t express the neuronal markers βIII-tubulin and NeuN (Fig 1A).

### Hipp-NSC express the stretch-activated ion channel Piezo1

The mRNA expression for mechanoreceptors Piezo 1/2, TRPV1/2, and TREK in Hipp-NSC cultured on Bioflex plates was assessed by qRT-PCR. Our data shows that Piezo1, TRPV1, and TREK mRNAs are detected in Hipp-NSC (Fig 1B). Double immunofluorescence analysis confirmed the presence of Piezo1 in nestin-positive Hipp-NSC (Fig 1C).

### Rapid stretch-induced LDH release from Hipp-NSC is not prevented by Piezo1 inhibition

**Fig 2:**
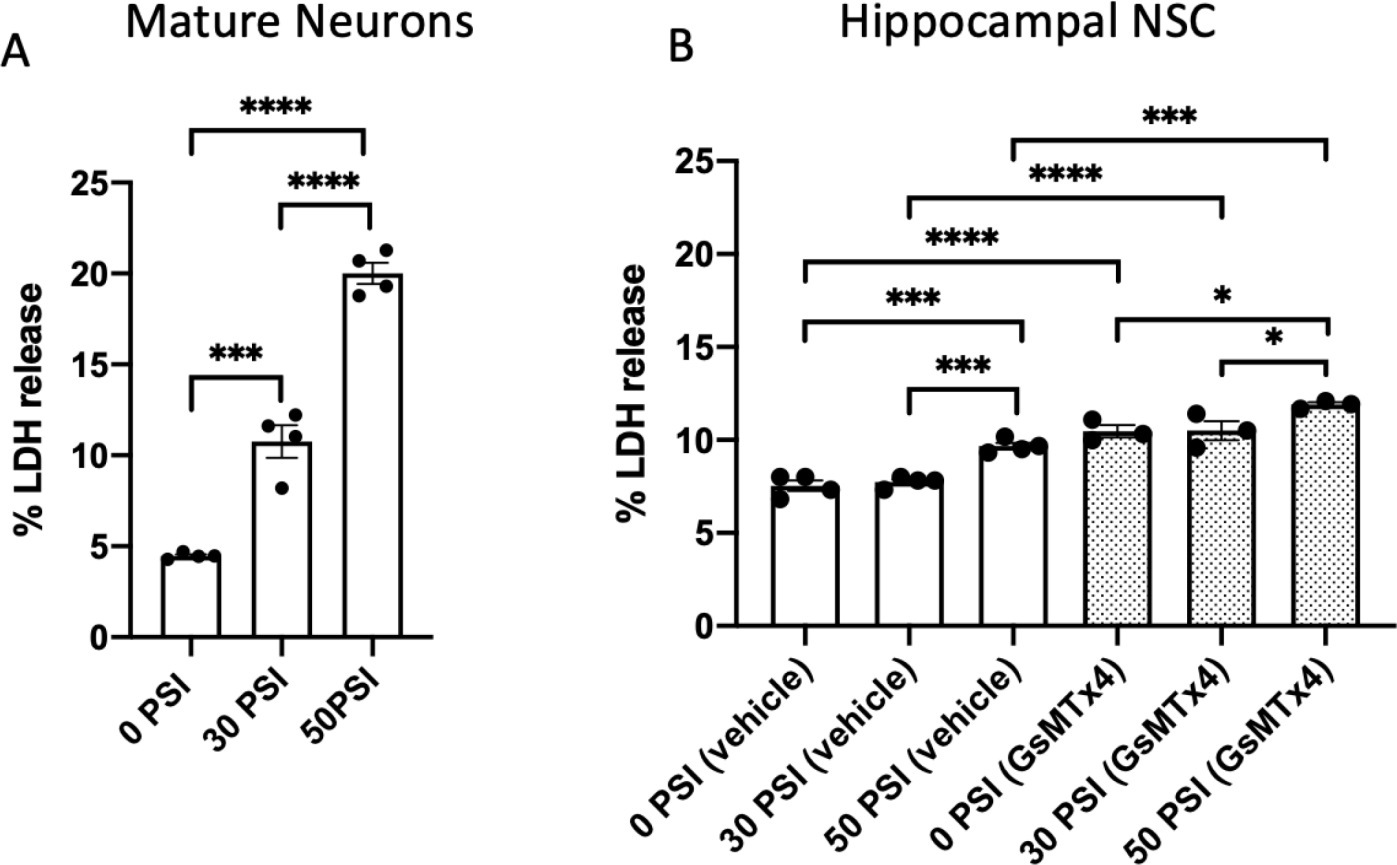
Stretch injury induces lactate dehydrogenase (LDH) release in Hipp-NSC. (A) LDH release from neurons stretched with 30 and 50 PSI. N = 4 independent experiments. (B) LDH release from Hipp-NSC stretched with 30 and 50 PSI PSI in the presence of vehicle or GsMTx4 (5μM). N = 3-4 independent experiments. Mean + /-SEM; *p < 0.05, **p < 0.01, ***p < 0.001 two-way analysis of variance (ANOVA) with Tukey’s post-hoc test.

To validate our injury paradigm, we subjected mature neurons, generated by differentiating Hipp-NSC, to a 30 PSI and a 50 PSI rapid stretch. We found a significant and pressure-dependent release of LDH from mature neurons, as expected and previously reported (Fig 2A). When Hipp-NSC were subjected to the same rapid stretch paradigm, we found that 30 PSI did not induce LDH release, while 50 PSI induced a significant release of LDH compared to both control Hipp-NSC (not-stretched) and Hipp-NSC stretched at 30 PSI (Fig 2B). Similarly, when Hipp-NSC were stretched in the presence of GsMtX4, an inhibitor of the mechanosensitive and stretch-activated ion channel Piezo1 (24), we found a significant increase in LDH release only in the 50 PSI stretch group as compared to the 30 PSI and no-stretch (0 PSI) groups (Fig 2B). Interestingly, we found a significant increase in LDH release when Hipp-NSC were incubated with GsMTx4 [24] compared to control Hipp-NSC (not-treated and not-stretched) (Fig 2B).

### Rapid stretch does not affect Hipp-NSC proliferation

**Fig 3:**
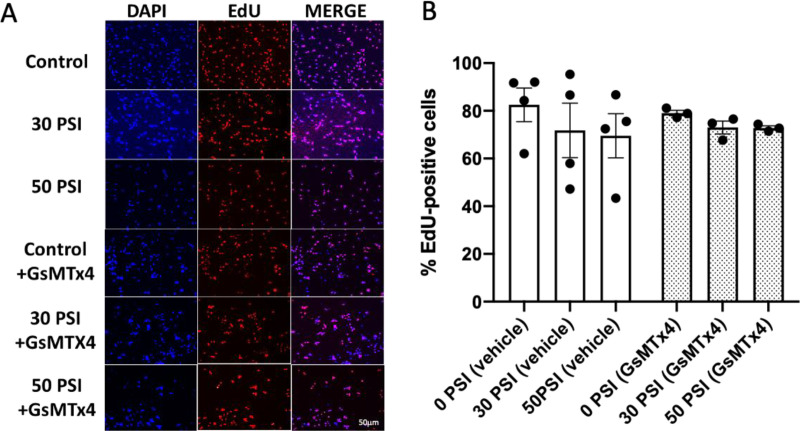
Stretch injury does not change Hipp-NSC proliferation. Representative images of EdU immufluorescence in Hipp-NSC. (B) Quantitative analysis of the proliferation of the neural stem cells (%EdU^+^ Hipp-NSC) stretched with 30 and 50 PSI in the presence of vehicle or GsMTx4 (5μM). N = 3-4 independent experiments. Mean + /- SEM; p > 0.05 two-way analysis of variance (ANOVA) with Tukey’s post-hoc test.

To study whether rapid stretch can affect Hipp-NSC proliferation, we added EdU to the culture media before the stretch injury and fixed the cells 24 hours later. In a parallel set of Hipp-NSC cultures, we added the inhibitor GsMTx4 to the media before stretch injury to inhibit Piezo1. Immunofluorescence analysis showed that rapid stretch, both in the presence and absence of GsMTx4, did not significantly change the number of EdU-positive Hipp-NSC (Fig 3).

### Piezo1 inhibition reduces glial differentiation and increases neuronal differentiation in Hipp-NSC subjected to rapid stretch

**Fig 4:**
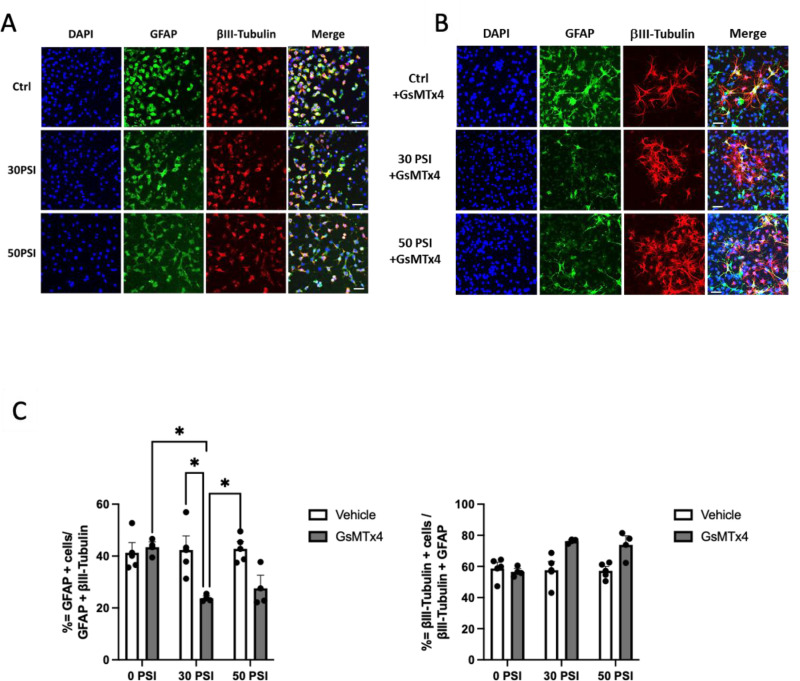
Piezo-1 inhibition directs neuronal differentiation in Hipp-NSC subjected to rapid stretch. (A-B) Representative images of stretched Hipp-NSC stained for βIII-Tubulin and GFAP, 7 days after differentiation + /- GsMTx4 (5μM); Ctrl: not stretched Hipp-NSC; (C) Quantification analysis of GFAP^+^ and βIII-Tubulin^+^ cells normalized to the total number of differentiated cells (GFAP^+ ^+ and βIII-Tubulin^+^/ DAPI); N = 3-4 independent experiments; *p < 0.05. Mean + /- SEM; two-way analysis of variance (ANOVA) with Tukey’s post-hoc test. Calibration bar = 50μm.

To analyze the effect of Piezo1 inhibition and rapid stretch on Hipp-NSC differentiation, proliferation media was immediately removed after stretch injury and replaced with differentiation media. After one week of differentiation, the cells were fixed and processed for immunofluorescence analysis of glial and neuronal markers (GFAP and βIII-Tubulin). Our data shows that when Hipp-NSC were subjected to rapid stretch in the absence of GsMTx4, there were no significant changes in the percentage of βIII-Tubulin positive cells or GFAP positive cells (Fig 4). In the presence of GsMTx4, there was a significant decrease in the percentage of GFAP-positive cells following the 30PSI stretch (Fig 4). However, in the presence of GsMTx4, the percentage of βIII-Tubulin positive cells did not significantly change (Fig 4).

### Piezo1 inhibition increases the expression of regulatory miRNAs in Hipp-NSC

**Fig 5:**
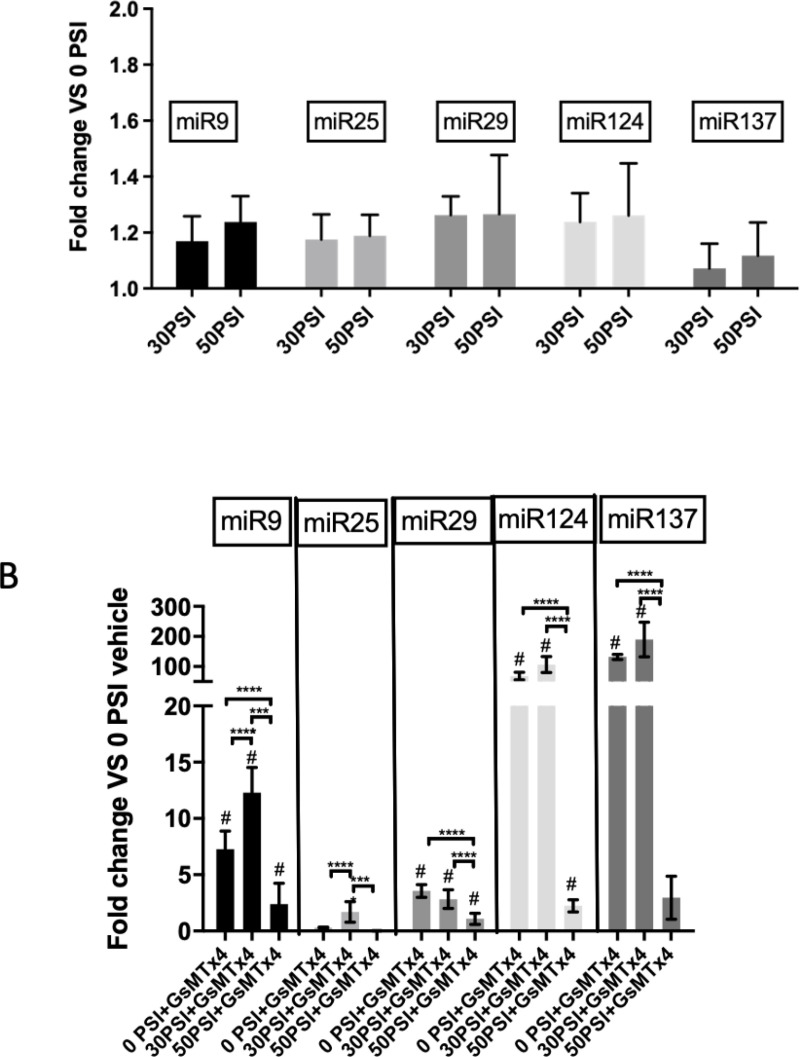
Piezo-1 inhibition increases the expression of regulatory miRNAs in Hipp-NSC. qRT-PCR analysis of the expression of miRNA of stretched Hipp-NSC as compared to not stretched Hipp-NSC. (B) qRT-PCR analysis of the expression of miRNA of stretched Hipp-NSC + GsMTx4 (5μM) as compared to not stretched Hipp-NSC. N=8; Mean +/- SEM; **p<0.001, ****p<0.0001, #p<0.0001; two-way analysis of variance (ANOVA) with Tukey’s post-hoc test.

To determine whether stretch injury can modulate the expression of specific miRNA known to regulate neurogenesis, we isolated RNA from stretched and unstretched NSC with and without GsMTx4. We performed qRT-PCR to assess the expression of miR9, miR25, miR29, miR124, miR137. Our data shows that the expression of regulatory miRNA is not significantly different 24 hours after rapid stretch compared to unstretched controls (Fig 5A). Interestingly, the presence of GsMTx4 significantly increased miRNA expression in Hipp-NSC (Fig 5B). Moreover, in the presence of GsMTx4, a 50 PSI stretch injury decreased miRNA expression compared to a 30 PSI stretch injury and uninjured cells with the inhibitor (Fig 5B).

qRT-PCR analysis of the expression of miRNA of stretched Hipp-NSC as compared to not stretched Hipp-NSC. (B) qRT-PCR analysis of the expression of miRNA of stretched Hipp-NSC + GsMTx4 (5μM) as compared to not stretched Hipp-NSC. N = 8; Mean + /- SEM; **p < 0.001, ****p < 0.0001, #p < 0.0001; two-way analysis of variance (ANOVA) with Tukey’s post-hoc test.

### Piezo1 knockdown reduces glial differentiation and increases neuronal differentiation in Hipp-NSC subjected to rapid stretch

**Fig 6:**
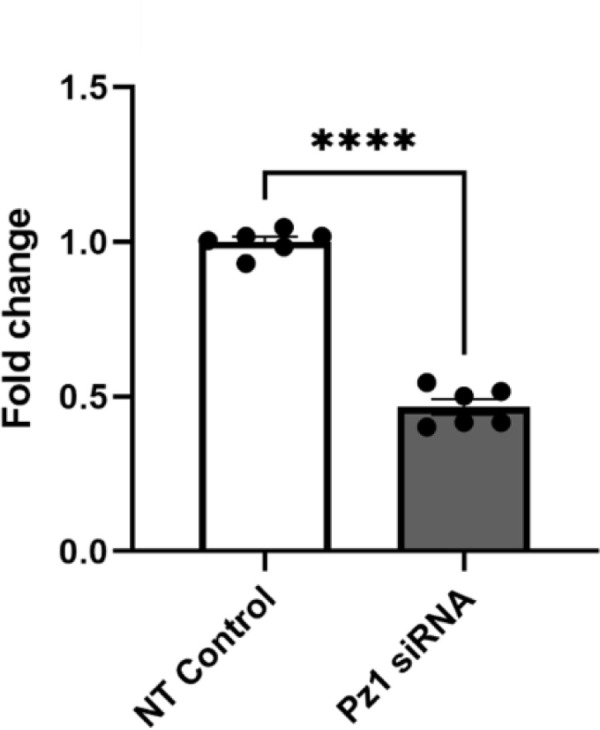
Quantitative RT-PCR of Piezo-1 after knockdown treatment in Hipp-NSC. qRT-PCR showing the fold change in Piezo1 expression following siRNA treatment in Hipp-NSC in the presence of Pz1 siRNA or NT Control. (N = 6) Mean + /- SEM; ****p < 0.0001 Two-sample t test.

**Fig 7:**
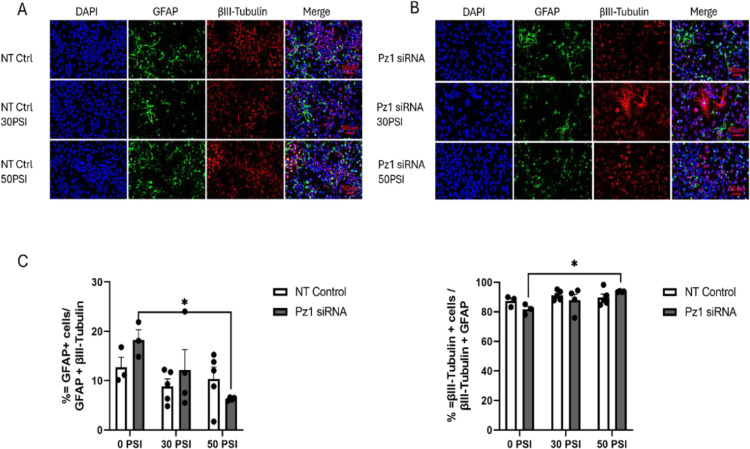
Piezo-1 knockdown directs neuronal differentiation in Hipp-NSC subjected to rapid stretch. (A-B) Representative images of stretched Hipp-NSC stained for βIII-Tubulin and GFAP, 7 days after differentiation + /- Pz1 siRNA (20 µ M); NT Ctrl (20 µ M); (C) Quantification analysis of GFAP^+^ and βIII-Tubulin^+^ cells normalized to the total number of differentiated cells (GFAP^+ ^+ and βIII-Tubulin^+^/ DAPI); Unstretched Pz1siRNA and NT Control N = 3, Pz1 siRNA 30 and 50 PSI N = 4, NT Control 30 and 50 PSI N = 5; *p < 0.05 Mean + /- SEM; two-way analysis of variance (ANOVA) with Tukey’s post-hoc test.

**Fig 8:**
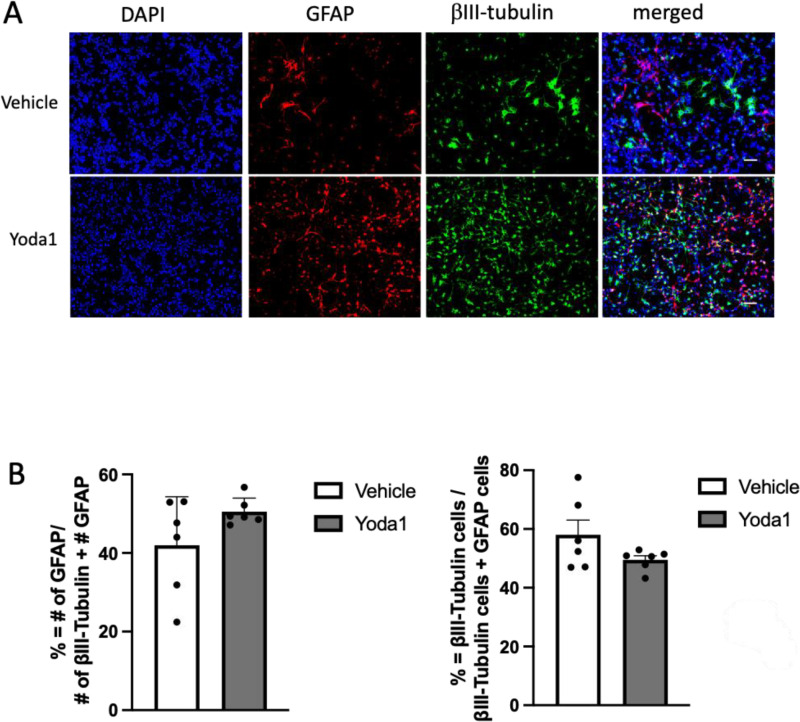
Non-mechanical activation of Piezo-1 does not change neuronal and glial differentiation of Hipp-NSC. (A-B) Representative images of Hipp-NSC treated with 10μM Yoda1 or vehicle and stained for βIII-Tubulin and GFAP 7 days after differentiation. (C) Quantification analysis of GFAP^+^ and βIII-Tubulin^+^ cells normalized to the total number of differentiated cells (GFAP^+ ^+ and βIII-Tubulin^+^/ DAPI). Mean + /- SEM. Two-sample t-test.

To isolate the effect Piezo1 has on Hipp-NSC differentiation, we utilized a Piezo1-targeting siRNA. Hipp-NSC were plated on BioFlex plates, treated with Piezo1 siRNA or Non-Targeting Control, and then collected for qRT-PCR. Our data shows that we achieved a 50% reduction in Piezo1 expression (Fig 6). To determine the effect Piezo1 knockdown has on Hipp-NSC differentiation, we treated the Hipp-NSC with Piezo1 siRNA or Non-Targeting siRNA 24 hours before stretch injury. After 7 days of differentiation, the cells were fixed and processed for immunofluorescence analysis of glial and neuronal markers (GFAP and βIII-Tubulin). The percentage of GFAP-positive cells and βIII-Tubulin positive cells did not significantly change following the Non-Targeting control treatment with and without stretch injury (Fig 7). However, the percentage of GFAP+ cells in the NT siRNA group was much less than normally observed in vehicle-treated Hipp-NSC from other experiments (Fig 4 and Fig 8). This could reflect a non-specific effect of siRNA independent of its target. The percentage of GFAP-positive cells and βIII-Tubulin-positive cells in the Piezo1 siRNA-treated cells without stretch injury did not change as compared to NT control. However, in Piezo1 siRNA-treated cells, we found a significant decrease in the percentage of GFAP-positive cells and a significant increase in βIII-Tubulin positive cells after the 50PSI stretch (Fig 7).

### Non-mechanical activation of Piezo1 does not change Hipp-NSC neuronal and glial differentiation

To further elucidate the role of Piezo1 in the mechanotransduction process of regulating Hipp-NSC differentiation, we utilized the specific agonist YODA1. Hipp-NSC were plated on BioFlex plates in proliferation media and treated with 10 μM YODA1 for 1 hour [25]. After 7 days of differentiation, the cells were fixed and processed for immunofluorescence analysis of glial and neuronal markers (GFAP and βIII-Tubulin). Non-mechanical activation of Piezo1 by Yoda1 did not significantly change the proportion of βIII-Tubulin positive cells and GFAP positive cells generated by Hipp-NSC differentiation (Fig 8).

## Discussion

Neural stem cells’ proliferation and lineage commitment are influenced by the mechanical properties of the environment in which they grow [26]. Specifically, soft surfaces promote NSC proliferation, while NSC cultured on stiffer surfaces are stimulated to differentiate [23]. This evidence highlights how biomechanics and mechano-transduction play a pivotal role in cell metabolism and fate specification [19]. Moreover, there are significant changes in the mechanical properties of the brain during traumatic brain injury (TBI). TBI is characterized by the disruption of normal brain function caused by an external mechanical force [27]. Specifically, brain tissue is stretched rapidly as impact, resulting in a sudden change in stiffness, which may explain, at least in part, reported changes in the abilities of NSC to proliferate and differentiate [28]. The stretch-activated ion channel Piezo1 has been shown to direct lineage specification in neural stem cells [21]. In this work, we have used an established *in vitro* stretch injury method [29,30] to evaluate the effect of a mechanical force applied directly to rat hippocampus neural stem cells (Hipp-NSC) in culture. To test the role of mechano-transduction signals in regulating Hipp-NSC proliferation and differentiation, we tested the effect of rapid stretch injury on Hipp-NSC in the absence or presence of Piezo1-targeting siRNA, GsMTx4, a peptide isolated from tarantula’s venom, known to act as Piezo1 antagonist and the Piezo1 small molecule agonist Yoda1 [31].

We first confirmed that Hipp-NSC maintains their stemness when grown attached to flexible plastic support, such as the stretchable flex bottom plate, and then confirmed the expression of Piezo1 on their surface. Two levels of rapid stretch were tested: 30 PSI and 50 PSI. Stretch-injured mature neurons showed a significant increase in the level of injury (LDH release) with both intensities in a dose-dependent manner. In contrast, Hipp-NSC showed a significant increase in LDH release only with the highest intensity (50 PSI). These results confirm the effectiveness of our injury model and suggest a better resistance to mechanical stress in Hipp-NSC than in mature neurons. Interestingly, GsMTx4 significantly increases LDH release in Hipp-NSC, suggesting that Piezo1 may be critical in maintaining cell viability under basal conditions. Indeed, the tonic activity of Piezo1 that allows Ca^2+^ influx essential for cell function has been previously shown in astrocytes and neurons [32,33]. Rapid stretch injury also induces an influx of Ca^2+^ ions that can lead to cytotoxicity. Interestingly, the treatment with GsMTx4 does not further increase the level of LDH after stretch until the highest intensity (50 PSI), confirming an essential role of Piezo1 in mechano-transduction and indicating a possible protective effect of the inhibition of Piezo1 during stretch injury.

In this study, we used pharmacological (GsMTx4) and siRNA strategies to inhibit Piezo1 and determine its role in regulating the differentiation and proliferation of Hipp-NSC after a mechanical insult (e.g., rapid stretch). We found that GsMTx4 alone, or Piezo1 downregulation using a selective siRNA, did not have a significant effect on the percentage of neurons or astrocytes generated by Hipp-NSC under basal conditions. However, when a rapid stretch was applied in the presence of Piezo1 inhibition or downregulation, there was an increase in the percentage of neurons generated and a decrease in the percentage of astrocytes generated from Hipp-NSC. Interestingly, our data shows that rapid stretch alone does not alter Hipp-NSC proliferation and differentiation. These results suggest a possible role for Piezo1 in regulating neurogenesis following TBI.

To better understand the role of Piezo1 in lineage commitment, we treated Hipp-NSCs with the Piezo1 agonist YODA1. YODA1 is a small molecule known to uniquely trigger Piezo1 by lowering its mechanical activation threshold and making Piezo1 more susceptible to opening following minor mechanical stimuli or spontaneously in the absence of mechanical induction [34]. We found that YODA1 activation of Piezo1 did not affect the neuronal or glial differentiation of Hipp-NSCs. This finding aligns with our data showing that mechanical stretch does not influence Hipp-NSC differentiation. It also suggests that Piezo1 may play an inhibitory role on other mechanoreceptors present in Hipp-NSCs (Fig 1B), further supporting its role in maintaining proper Hipp-NSC lineage specification in response to mechanical insults such as TBI.

Finally, we investigated the effect of rapid stretch and Piezo1 inhibition on the expression of regulatory microRNAs (miRNAs) known to regulate neurogenesis [35,36,37]. While we found that rapid stretch did not significantly change the relative expression of neurogenesis regulatory miRNAs in Hipp-NSC, Piezo1 inhibition significantly increased their expression. These results align with other reports showing that tonic activation of mechanoreceptors, such as Piezo1, plays a critical role in regulating cell activity [20]. Interestingly, when Piezo1 is inhibited, a 30 PSI rapid stretch increases miRNA expression in Hipp-NSC while a 50 PSI rapid stretch significantly downregulates them compared to not-stretched controls. This could be explained by the activation of Piezo1-independent mechanisms with increased stretch intensity. However, further studies are necessary to characterize these observations fully.

In conclusion, our results align with other studies showing that mechanical changes, induced either by pharmacological inhibition of mechanoreceptors or changes in substrate stiffness, induce neuronal differentiation of NSC [21,38,39], and confirm the critical role of the stretch-activated ion channel Piezo1 in the transduction of the mechanical stress that affects neurogenesis during TBI. The limitation of this study is that we focused on the role of Piezo1, but other mechanoreceptors are expressed on the NSC surface (Fig 1B). For this reason, the mechanical stimuli from the environment may result from the synergic contribution of the different mechanoreceptors and not of Piezo1 alone. Further experiments are necessary to better understand the molecular mechanism of mechano-transduction signals in Hipp-NSC during stretch injury.

## Methods

### Ethics statement

All experiments reported in this manuscript were conducted *in vitro* using hippocampal neural stem cells obtained from Millipore Sigma, a commercial source. Since this study did not involve the use of vertebrate animals, it was not subject to review by the UTMB Institutional Animal Care and Use Committee.

### Neural stem cell culture

Adult rat hippocampal neural stem cells (Hipp-NSC, Millipore Sigma) were cultured in suspension as neurospheres in proliferating media (DMEM/F12 supplemented with 2% B27 without vitamin A, 20ng/mL FGF, 1% L-glutamine, and 1% antibiotic/antimycotic) according to Manufacturer’s instructions. For the stretch injury, Hipp-NSC were plated on polyornithine and laminin-coated plates with a flexible silicone bottom (BioFlex® Culture Plates, Flex International Corporation) at a density of 2 x 10^5^ cells/ 10 cm^2^. For differentiation, Hipp-NSC were cultured in differentiation media (DMEM/F12 supplemented with 2% B27 with vitamin A, 1% L-glutamine, 1% FBS, and 1% antibiotic/antimycotic) for seven days, changing the media every other day.

### Poly-Ornithine-Laminin coating

Poly-L-ornithine (Sigma-Aldrich) stock solution was reconstituted in sterile water at a concentration of 10 mg/mL, aliquoted, and stored at -20ºC. Laminin (Millipore) was reconstituted in sterile 1X PBS at a concentration of 6 µg/mL. The poly-L-ornithine stock solution was diluted to 10 µg/mL in sterile water, 1.5 mL was added to each well of the 6-well stretch plates, and 0.5 mL was added to each chamber slide well. The plates and slides were incubated at RT overnight. The following day, the plates and slides were rinsed twice with sterile water, 1.5 mL of laminin was added to each well of the 6-well stretch plates, and 0.5 mL was added to each chamber slide well. The 6 well stretch plates and chamber slides were kept at 4ºC until used.

### Stretch injury

Twenty-four hours after siRNA treatment, the transfection media was replaced with fresh differentiation media. Hipp-NSC was stretched using a Cell Injury Controller II (Earl Ellis Laboratory, Virginia Commonwealth University, Richmond, VA) connected to a nitrogen tank. For the stretch injury, the Hipp-NSC received a 30 or 50 PSI injury with a 50ms duration. Hipp-NSC was allowed to differentiate for 7 days, receiving fresh media every other day. Hipp-NSC that did not receive stretch injury were collected for RNA extraction and qRT-PCR analysis.

### Lactate dehydrogenase (LDH) assay

To determine viability, immediately after rapid-stretch injury 50μl of media was mixed with 50μl of tetrazolium salt (INT) per the manufacturer’s protocol (Thermo Fisher). The conversion of INT into a red formazan product was quantified by measuring the absorbance at 490nm using a GloMax®-Multi+ Detection System (Promega).

### Proliferation assay

EdU (5-ethynyl-2’-deoxyuridine) was used to mark replicated DNA of proliferating cells. EdU was diluted from the stock solution (10mM) to a final concentration of 10 µ M in culture medium and added to the cells. The cells were washed in PBS 24 hours following stretch injury and fixed with ice-cold 100% methanol on ice for 20 minutes. The cells were washed twice with 3% BSA in PBS to remove the fixative. The cells were permeabilized with a 0.5% Triton-X in PBS solution for 20 minutes, then washed twice with 3% BSA in PBS. The terminal alkyne group of EdU was detected through its cycloaddition “click” reaction catalyzed by copper with fluorescent azides per the manufacturer’s instructions (Invitrogen). At the end of the reaction, the cells were washed once with 3% BSA in PBS at the nuclei were stained with Hoechst 33342 (5µg/ml) for 30 minutes. Images were taken using a confocal microscope (Olympus 4A43956 FV1200) supported with FluoView 4.0 software.

### Cell treatment with the Piezo1 antagonist GsMTx4 and Piezo1 agonist yoda1

GsMTx4 (Alamone Labs), an antagonist of the mechanoreceptor Piezo1, was dissolved in PBS to a final concentration of 1 mM. Hipp-NSC were treated for 30 minutes before stretch injury by diluting GsMTx4 in the media to a 5 µ M concentration as previously reported^27^ and maintained in the media for 24 hours following the stretch injury. The next day, the cells were either fixed for EdU analysis or lysed in Triazol for total mRNA collection. For differentiation, 24 hours after stretch injury, the media containing GsMTx4 was replaced with fresh differentiating media without the antagonist and allowed to differentiate for 7 days.

Yoda1 (Millipore), a small molecule selective agonist of Piezo1, was dissolved in DMSO to a final concentration of 42.22 mM. Hipp-NSC were treated for 1 hour with 10 μM Yoda1 in proliferation media. The concentration of Yoda1 was chosen based on prior published reports [25]. At the end of the incubation, the media was replaced with fresh differentiating media and Hipp-NSC were allowed to differentiate for 7 days.

### Cell treatment with Piezo1 siRNA

5X siRNA Buffer (Horizon) was diluted in sterile RNase-free water to 1X 5 nmol ON-TARGETplus Non-Targeting Pool, and 5 nmol ON-TARGETplus Rat Piezo1 siRNA (Horizon) was diluted in 1X siRNA buffer to 20 µ M. For the transfection, Non-Targeting Pool and Rat Piezo1 siRNA were diluted in Opti-MEM and Lipofectamine™ 3000 (Thermo Fisher) to a final concentration of 100nM and added to Hipp-NSC in proliferation media (DMEM/F12 supplemented with 2% B27 without vitamin A, 20ng/mL FGF, and 1% L-glutamine) [40].

### Cell immunofluorescence

Hipp-NSC were washed with 1X PBS and then fixed in ice-cold 10% formalin for 15 minutes at RT. The wells were washed with 1X PBS 3 times for 5 minutes, then blocked and permeabilized for 1 hour in a solution containing 10% normal serum, 0.3% Triton X-100, and 0.3M glycine in PBS.

To confirm Piezo1 expression on Hipp-NSC, immunostaining was performed using the following antibodies: rabbit anti-Piezo1 (1:200, Alomone Lab) and mouse anti-Nestin antibody (1:1000. Millipore).

To determine differentiation, stretched Hipp-NSC were incubated overnight with rabbit anti-GFAP (1:1000, Dako) and mouse anti-βIII-Tubulin antibody (1:1000, Promega). The following day, the cells were washed 2 times for 5 minutes in 1X PBS, then blocked a second time for 30 minutes at RT. The cells were then incubated for 1 hour at RT in Alexa-conjugated antibodies diluted in 1.5% normal serum. The cells were washed 3 times for 7 minutes in 1x PBS, then incubated for 10 minutes with DAPI (1:1000 Thermo Fisher). The cells were rinsed with chilled 1X PBS and then mounted on a microscope slide. Images were taken using a fluorescence microscope BZ-X710 (Keyance) supported by BZ-X analyzer software (Keyance). Images were quantified using Image J software. The slides were stored in the dark at 4ºC.

### Cell count

To measure EdU incorporation, EdU-positive cells were counted from five different fields per well for each group. The ratio of the proliferating cells was obtained by dividing the EdU-positive cells from the total number of cells present in the field denoted by DAPI.

To measure differentiation, GFAP+ cells and βIII-Tubulin+ cells were counted from five different fields per well for each group. The ratio of differentiation was calculated by dividing the number of positive cells by the total number of cells present in the field denoted by DAPI.

Images were taken with a confocal microscope (Olympus IX83) supported with FluoView 4.0 software and a fluorescence microscope BZ-X710 (Keyance) supported by BZ-X analyzer software (Keyance) and quantified using Image J software.

### Protein isolation and western blotting analysis

For total protein extraction, unstretched and stretched Hipp-NSC were cultured in differentiating media for 7 days. The cells were collected by ultracentrifugation, washed in 1X PBS, and lysed using RIPA lysis buffer containing protease and phosphatase inhibitors (Thermo Fisher) for 10 minutes on ice. The samples were pelleted by centrifugation (15 minutes 14,000 x g), and the supernatant was aliquoted and stored at -20ºC until used. Total protein concentration was determined using the BCA assay kit according to the manufacturer’s protocol (Thermo Fisher).

Proteins (25 µg per sample) were resolved by SDS-PAGE using precast 4–20% gradient gels (Biorad) and transferred to polyvinylidene fluoride (PVDF) membranes. The membranes were incubated overnight at 4ºC with the following antibodies: rabbit anti (PCNA) (1:1000, Abcam); mouse anti-βIII-Tubulin antibody (1:1000, Sigma); mouse anti-Sox2 (1:1000, Cell Signaling); mouse anti-NeuN (1:1000, Millipore); mouse anti-Nestin antibody (1:1000, Millipore). The quantification was performed with Image lab software (BioRad), and HRP conjugated Glyceraldehyde 3-phosphate dehydrogenase (GAPDH) (1:3000. Thermo Fisher) was used as a normalizer.

### RNA isolation

According to the manufacturer’s instructions, the total RNA was isolated using the Quick-RNA Miniprep Kit (Zymo Research). Briefly, cultured Hipp-NSC were lysed at 0h and 24h after stretch injury with RNA lysis buffer (400 µ L/ 5x10^6^ cells). The lysed cells were transferred to a column provided by the kit and centrifuged at 13,000 x g for 30 seconds. An equal volume of 100% ethanol was added to the flowthrough, mixed well, transferred to another column, and centrifuged as before. The column was then washed with RNA Wash Buffer, followed by a 15-minute incubation with DNase I and DNA Digestion Buffer. RNA Prep Buffer was added to the column and centrifuged, followed by 2 washes of RNA Wash Buffer. Finally, DNase/RNase- Free water was added to the column and centrifuged to elute the complete RNA. RNA concentration was quantified using a NanoDrop ONE (Thermo Fisher) and immediately stored at -80ºC.

### RNA retrotrvanscription

Total RNA (200 mg) was used for the retrotranscription of the pool of mRNA using the iScript cDNA Synthesis Kit (BioRad) performed using a thermocycler following these steps: priming reaction one cycle for 5 minutes at 25ºC, reverse transcription for 20 minutes at 46ºC, RT inactivation for 1 minute at 95ºC, then hold at 4ºC. The results of the reaction were stored at -20ºC until further use.

### microRNA retrotranscription

Total RNA (10 µg) was used to retrotranscribe the pool of miRNA using the TaqMan Advanced miRNA Assay Kit (Thermo Fisher), which was performed using a thermocycler following these steps: First, poly (A) tailing reaction for one cycle for 45 minutes at 37ºC, then 10 minutes at 65ºC, and hold at 4ºC. Second, ligation reaction for one cycle for 15 minutes at 42ºC, then 5 minutes at 85ºC, and hold at 4ºC. Third, reverse transcription reaction for one cycle for 15 minutes at 42ºC, then 5 minutes at 85º, and hold at 4ºC. Fourth, miR-Amp reaction for 1 cycle for 5 minutes at 95ºC, two-step PCR reaction with 14 cycles each for 3 seconds at 95ºC and 30 seconds at 60ºC, then 1 cycle for 10 minutes at 99ºC and hold at 4ºC. The results of the reaction were stored at -20ºC until further use.

### Total RNA retrotranscription

Total RNA (10 µg) was used to retrotranscribe the pool of siRNA using the TaqMan Advanced miRNA Assay Kit (Thermo Fisher), which was performed using a thermocycler following these steps: First, poly (A) tailing reaction for one cycle for 45 minutes at 37ºC, then 10 minutes at 65ºC, and hold at 4ºC. Second, ligation reaction for one cycle for 15 minutes at 42ºC, then 5 minutes at 85ºC, and hold at 4ºC. Third, reverse transcription reaction for one cycle for 15 minutes at 42ºC, then 5 minutes at 85º, and hold at 4ºC. Fourth, miR-Amp reaction for 1 cycle for 5 minutes at 95ºC, two-step PCR reaction with 14 cycles each for 3 seconds at 95ºC and 30 seconds at 60ºC, then 1 cycle for 10 minutes at 99ºC and hold at 4ºC. The results of the reaction were stored at -20ºC until further use.

### Quantitative Real-Time PCR (qRT-PCR)

qRT-PCR was performed on a MX3000P system (Stratagene, Santa Clara, CA) using Taqman reagents from Applied Biosystems (Foster City, CA). GAPDH was chosen as the reference gene due to its stable expression across the conditions tested.

### miRNA qRT-PCR

To perform miRNA qRT-PCR, a 20 µ L PCR reaction was made by mixing 10 µ L of 2X Fast Advanced MasterMix, 2 µ L of the product of the retrotranscription, 1 µ L of predesigned Taqman Advanced miRNA assay primers from Applied Biosystems (Foster City, CA) and 6 µ L of nuclease-free water. The thermal profiler setup used for the PCR reaction was 1 cycle for 2 minutes at 50ºC, 1 cycle for 20 seconds at 95ºC, 40 cycles for 3 seconds at 95ºC, and 40 cycles for 30 seconds at 60ºC. All data from the PCR was collected and analyzed using LightCycler® 96 software, and the ΔΔCT fold changes were determined using the calibrator (GAPDH).

### Piezo-1 QRT-PCR

To perform Piezo-1 qRT-PCR, a 20 µ L PCR reaction was made by mixing 10 µ L of 2X Fast Advanced MasterMix, 2 µ L of the product of the retrotranscription, 1 µ L of predesigned Taqman Advanced Piezo1 and GAPDH assay primers from Applied Biosystems (Foster City, CA) and 6 µ L of nuclease-free water. The thermal profiler setup used for the PCR reaction was 1 cycle for 2 minutes at 50ºC, 1 cycle for 20 seconds at 95ºC, 40 cycles for 3 seconds at 95ºC, and 40 cycles for 30 seconds at 60ºC. All data from the PCR was collected and analyzed using LightCycler® 96 software, and the ΔΔCT fold changes were determined using the calibrator (GAPDH).

### Statistical analysis

Data is expressed as mean + /- SEM. Analysis of variance (ANOVA) followed by multiple comparisons post-hoc Tukey’s tests were performed using GraphPad 9 Prism software.

Differences were considered significant when p < 0.05

## Supporting information

S1 raw image Uncutted western blotting membranes incubated with the specified antibodies. (PDF)

S2 raw data Underlying raw data used to generate the graphs in our manuscript. (XLSX)
