# Supplementary material for: Mechanosensitive ion channel Piezo1 modulates the response of rat hippocampus neural stem cells to rapid stretch injury
Source: PLoS One. 2025 May 13;20(5):e0323191. doi: 10.1371/journal.pone.0323191 (PMC12074584; doi:10.1371/journal.pone.0323191)
Supplement: S1 raw image — (PDF) [file pone.0323191.s001.pdf]

Figure 1 A_Raw data

Blot1_Stain-free_loading

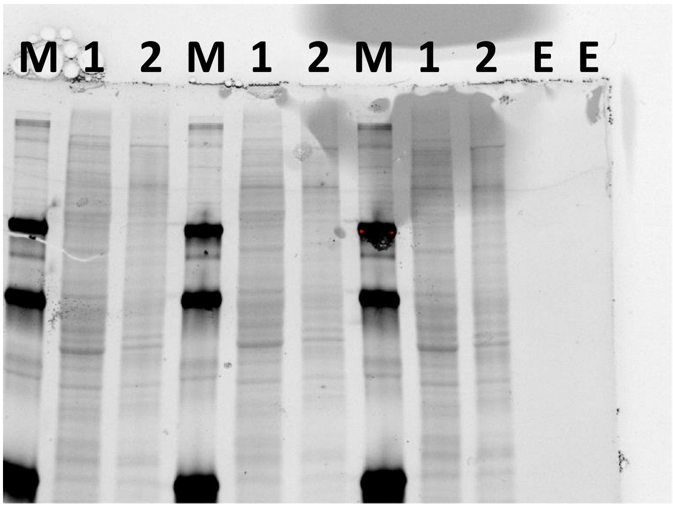

M: Marker; 1: Hipp-NSCs; 2: Brain extract; E: Empty

Blot2_Stain-free_loading

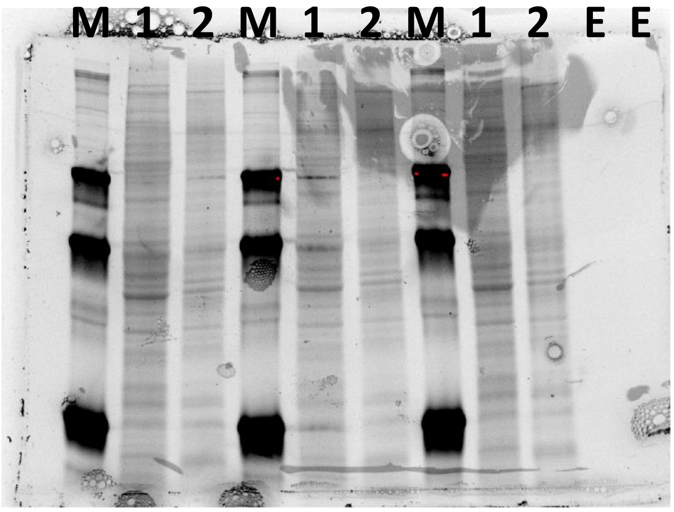

M: Marker; 1: Hipp-NSCs FlexPlate; 2: Brain extract; E: Empty

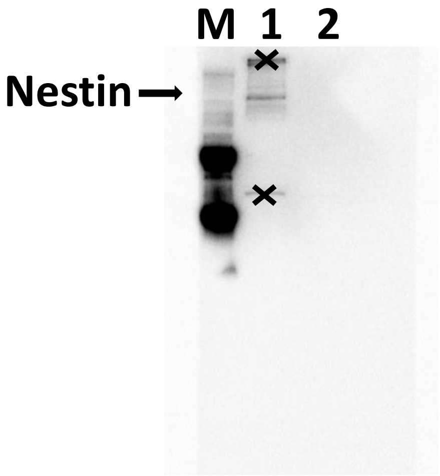

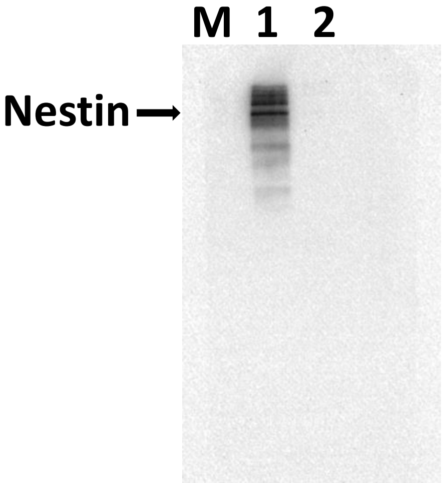

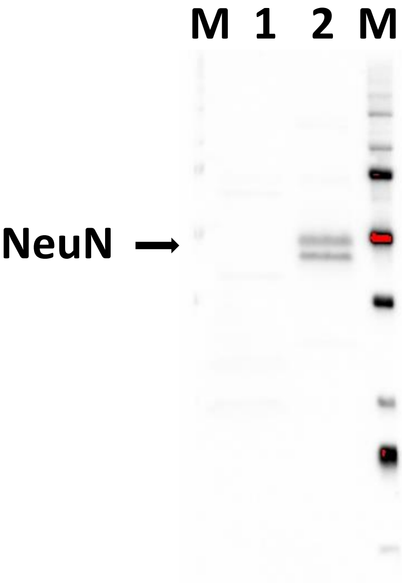

M: Marker; 1: Hipp-NSCs; 2: Brain extract

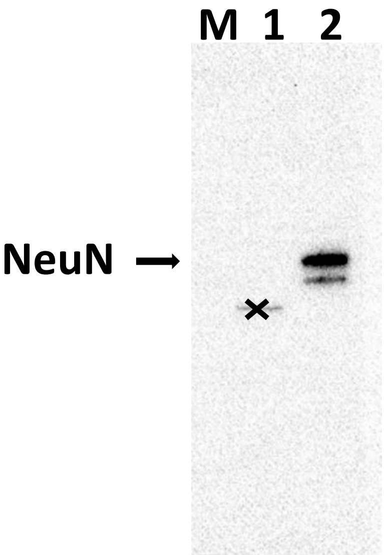

M: Marker; 1: Hipp-NSCs FlexPlate; 2: Brain extract

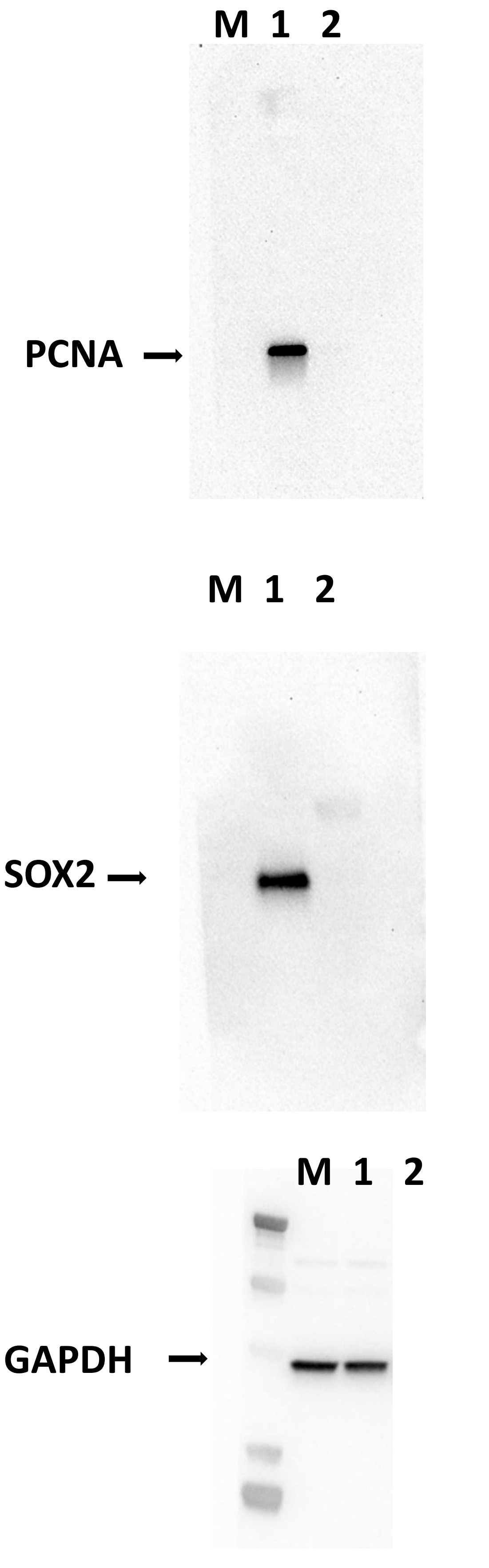

M: Marker; 1: Hipp-NSCs; 2: Brain extract

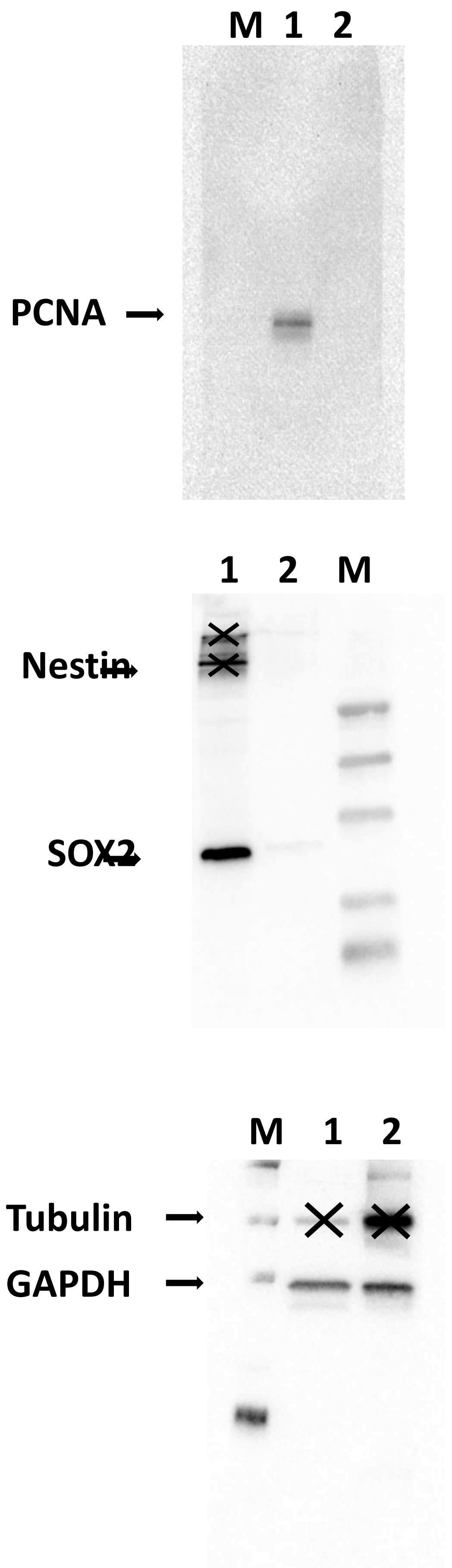

M: Marker; 1: Hipp-NSCs FlexPlate; 2: Brain extract
